# Toward a causal model of chronic back pain: Challenges and opportunities

**DOI:** 10.3389/fncom.2022.1017412

**Published:** 2023-01-11

**Authors:** J. Russell Huie, Rohit Vashisht, Anoop Galivanche, Constance Hadjadj, Saam Morshed, Atul J. Butte, Adam R. Ferguson, Conor O'Neill

**Affiliations:** ^1^Department of Neurosurgery, Brain and Spinal Injury Center, Weill Institutes for Neurosciences, University of California, San Francisco, San Francisco, CA, United States; ^2^San Francisco Veterans Affairs Healthcare System, San Francisco, CA, United States; ^3^Bakar Computational Health Sciences Center, University of California, San Francisco, San Francisco, CA, United States; ^4^Department of Orthopaedic Surgery, University of California, San Francisco, San Francisco, CA, United States; ^5^Departments of Orthopaedic Surgery and of Epidemiology, University of California, San Francisco, San Francisco, CA, United States

**Keywords:** causal (structural) model, back pain, data science, clinical trials, pain

## Abstract

Chronic low back pain (cLBP) afflicts 8. 2% of adults in the United States, and is the leading global cause of disability. Neuropsychiatric co-morbidities including anxiety, depression, and substance abuse- are common in cLBP patients. In particular, cLBP is a risk factor for opioid addiction, as more than 50% of opioid prescriptions in the United States are for cLBP. Misuse of these prescriptions is a common precursor to addiction. While associations between cLBP and neuropsychiatric disorders are well established, causal relationships for the most part are unknown. Developing effective treatments for cLBP, and associated co-morbidities, requires identifying and understanding causal relationships. Rigorous methods for causal inference, a process for quantifying causal effects from observational data, have been developed over the past 30 years. In this review we first discuss the conceptual model of cLBP that current treatments are based on, and how gaps in causal knowledge contribute to poor clinical outcomes. We then present cLBP as a “Big Data” problem and identify how advanced analytic techniques may close knowledge gaps and improve clinical outcomes. We will focus on causal discovery, which is a data-driven method that uses artificial intelligence (AI) and high dimensional datasets to identify causal structures, discussing both constraint-based (PC and Fast Causal Inference) and score-based (Fast Greedy Equivalent Search) algorithms.

## Introduction

Chronic low back pain (cLBP) is a debilitating syndrome that creates a high burden for both individuals and societies. For example, back pain is one of the most common reasons for medical visits (Deyo, 2015) and chronic low back pain (cLBP) is the leading global cause of disability (Hartvigsen et al., 2018).

The biopsychosocial model is the dominant framework for understanding chronic low back pain (cLBP) (Hartvigsen et al., 2018). According to this model cLBP results from an interplay between noxious stimuli in peripheral tissues and complex pain signal processing in the central nervous system (CNS) that is influenced by psychological and social factors. While widely accepted, the results from treatments inspired by the biopsychosocial model of cLBP model are marginal. These therapies—such as self-management, physical and psychological therapies, and non-opioid medicationshave been tested in numerous randomized controlled trials (RCTs) conducted over several decades. Treatment effects are typically small, with even the best treatments improving pain by only two points on a 0–10 visual analog scale (VAS) (Chou et al., 2016).

In order to improve outcomes for cLBP it is important to understand why current treatments are generally ineffective in RCT's. This may be because of heterogeneity of treatment effect (HTE). RCT's estimate an average treatment effect (ATE) for an intervention (Kent et al., 2019). The clinically relevant question, though, is what the effect of an intervention is at a subgroup level, among individuals with shared characteristics that may affect treatment response (Kent et al., 2019). HTE is a particular concern in assessing the effects of cLBP treatments, given heterogeneity among cLBP patients (Li et al., 2021). Another reason for the failure of cLPB treatments may be that they do not target the causes of cLBP. Many cLBP treatments are based on the assumption that associations between risk factors and cLBP are causal. For example, cLBP patients often have high fear-avoidance (they avoid activity because they fear pain the pain that results) (Linton and Shaw, 2011). The Fear-Avoidance model of cLBP assumes that fear-avoidance leads to cLBP, due to disuse and deconditioning (Linton and Shaw, 2011). It is entirely possible, however, that cLBP causes fear avoidance; i.e., the association is due to reverse causation. Many cLBP treatments target fear-avoidance, with at best mixed success, and there is evidence that changes in fear-avoidance may not be responsible for observed treatment effects (Sisco-Taylor et al., 2021). Better understanding of cLBP causes and how treatments impact those causes is needed to guide improvements to existing treatments and develop new treatment strategies.

### Chronic low back pain as a big-data problem

While the term “big data” has become a popular buzzword within disciplines with high data volume, big data is actually defined by the three vs: *volume, velocity, and variety*. Any one of these three elements can present a unique set of opportunities and challenges. For cLBP, as with related fields such as neurotrauma, *variety* may pose a bigger problem than *volume* (Huie et al., 2018).

In 1987, well before the term “big data” was popularized, Gordon Waddell sought to integrate the complex variety of factors believed to influence cLBP into a single model (Waddell, 1987). Waddell's model was based on (a) the principles of the biopsychosocial model of illness, at that time applied primarily in the behavioral sciences, (b) the gate control theory of pain developed by Melzak and Wall (which introduced the concept of neuromodulation) (Melzack and Wall, 1965) and (c) evidence that cLBP was associated primarily with psycho-social factors rather than spinal pathology. According to Waddell's model cLBP, including pain related disability, results from an interplay between noxious stimuli in peripheral tissues and complex pain signal processing in the central nervous system (CNS) that is influenced by psychological and social factors (Waddell, 1987). Waddell's conceptual model is now the dominant framework for both researching and treating cLBP (Hartvigsen et al., 2018).

**Figure 1:**
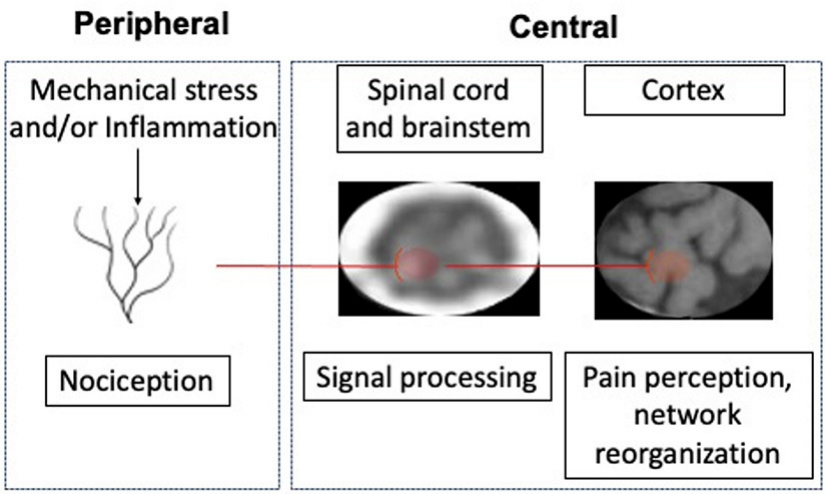
Biological factors associated with chronic low back pain (cLBP).

A large body of research supports the biopsychosocial model, for the most part as originally formulated by Waddell. Biological factors associated with pain can affect either the periphery or the central nervous system (Figure 1). In the periphery specialized neurons (nociceptors) in the discs, joints, fascia and muscles can be activated by mechanical or inflammatory stimuli. While age related spinal degeneration is not associated with chronic low back pain (Brinjikji et al., 2015), other potential peripheral nociceptive triggers are. These include vertebral endplate abnormalities, disc extrusions (Hartvigsen et al., 2018), altered biomechanics (Meir et al., 2019), poor muscle quality (Kalichman et al., 2017), heavy loading or twisting (Urban and Fairbank, 2020), and trigger points (Chiarotto et al., 2016). Interactions between factors are likely, and many may have a common nociceptive mechanism, namely inflammation (Khan et al., 2017). Centrally, signals from nociceptors are processed by neurons in the dorsal horn of the spinal cord and brainstem before activating cortical neurons, which results in pain perception. Modulation of nociceptive signals in the CNS can include facilitation and/or inhibition. Chronic pain is often accompanied by central sensitization, characterized by persistent facilitation of nociceptive signals (Williams, 2018). While Waddell's original module included neuromodulation, more recently, another CNS abnormality associated with cLBP has been described, namely anatomical and functional reorganization of the neocortex (i.e., limbic system) (Apkarian, 2019). This reorganization may be an indication that pain has been decoupled from nociception and has become a primarily cortical phenomenon (Apkarian, 2019). Associations between psychological and social factors and cLBP (Maher et al., 2017; Hartvigsen et al., 2018) are well established. There are a number of other variables associated with pain which may interact with the principal elements of the biopsychosocial model, including age (Wong et al., 2017), gender (Fehrmann et al., 2018), race (Meints et al., 2018), culture (Henschke et al., 2016), and co-morbidities (Rundell et al., 2017).

In 2018 the NIH established the Helping to End Addiction Long-term (HEAL). Initiative, a trans-agency effort to speed scientific solutions to stem the national opioid public health crisis. Recognizing the role of inadequately treated cLBP in the opioid crisis HEAL funded the Back Pain Consortium Research Program (BACPAC), which subsequently awarded 13 grants, totaling $130.8 million, to BACPAC research centers. The goal of BACPAC is to develop effective, individualized treatments for cLBP patients, based on the biopsychosocial model. Individual BACPAC centers are using a variety of methods to contribute to this goal, including technology development (seven studies), longitudinal cohort studies focused on deep phenotyping (three studies, two being conducted at UCSF), single endpoint randomized clinical trials (RCT's, two studies) and RCT's with multiple endpoints and sequential treatment assignments (SMART trials, two studies). Common data elements have been established that include measurements from several domains associated with cLBP: biological, psychological, and social factors; pain experience; demographics; and co-morbidities (Table 1). Tools used to collect these measurements include surveys, traditional and novel imaging techniques, quantitative sensory testing (QST), physical examination, sophisticated biomechanical testing devices, wearable sensors, biospecimen analysis, and extraction of electronic health record (EHR) data.

**Table 1:** Domains and common elements associated with chronic low back pain.

**Domain**	**Data elements**
Peripheral biological	Spine anatomy, muscle quality, endplate damage, tissue inflammation, disc biochemistry, serum/blood/tissue biomarkers, posture, kinematics, tissue loads, movement biomechanics, physical capacity and performance.
Central biological	Pain thresholds, pain modulation, aberrant pain processing, brain structure, brain function, interoceptive awareness
Psychological	Anxiety, depression, fear avoidance, catastrophizing, coping, acceptance, self-efficacy, stress, expectancy, beliefs in pain control
Social	Adverse life events, income, education, employment, financial strain, perceived discrimination, neighborhood, social isolation,
Demographics	Age, race, gender, ethnicity
Co-morbidities	Medical illnesses, chronic overlapping pain conditions, substance use
Pain experience	Intensity, duration, location, quality, interference (physical and social), sleep, fatigue, cognition

The types of data include structured data from surveys, examinations, electronic health records, and interpretation of diagnostic tests. Additionally, there is unstructured data from biomedical images, electronic health records, biomechanical assessments and wearable sensors.

The BACPAC single end-point RCT's will define the ATE for the treatments being studied, while the two SMART studies will determine optimal sequencing of treatments (Kidwell et al., 2018) based on individual patient characteristics. Those results can only be generalized to the specific treatments studied, under the trial conditions. However, combining data from the BACPAC research centers, facilitated by common data elements, would create a comprehensive multi-modal dataset that could be used to generate additional knowledge about cLBP. Given both the variety and volume of data, advanced analytic methods using AI tools, would be needed to do so. Among the tools that could be deployed are unsupervised learning methods, to identify clusters, and classification algorithms, to build predictive models. These methods define associations between features in the dataset, but do not reveal causal relationships. However, by applying recent innovations in data analysis this dataset could also be used to answer causal questions, such as *how do cLBP treatments work, and who do they work for?*. These innovative methods, collectively referred to as causal discovery methods, would couple data-driven approaches with principles of causal inference to unravel the complex relationships underlying cLBP.

### Causal inference

While there are a number of causal inference methods (Hernán et al., 2019) they share a common property- they answer “what if”, also known as counterfactual, questions (Bours, 2020). Counterfactual reasoning is a hallmark of human thought that engages a number of different brain regions to envision how an event might have unfolded differently under a different set of circumstances (i.e., circumstances that are counter-to-the-fact of what actually existed) (Hoeck et al., 2015; Bours, 2021; Raita et al., 2021). For example, faced with a bad result on an exam, one might reason that studying harder would have given a better result. Since going back in time is not possible there is no way to prove this, but causal reasoning leads to adaptive behavior (Hoeck et al., 2015); i.e., studying harder next time. Causal inference methods answer counterfactual questions, by formalizing counterfactual reasoning with mathematical notation (Hernán et al., 2019), allowing causal effects to be quantified using statistical methods (Bours, 2020).

**Figure 2:**
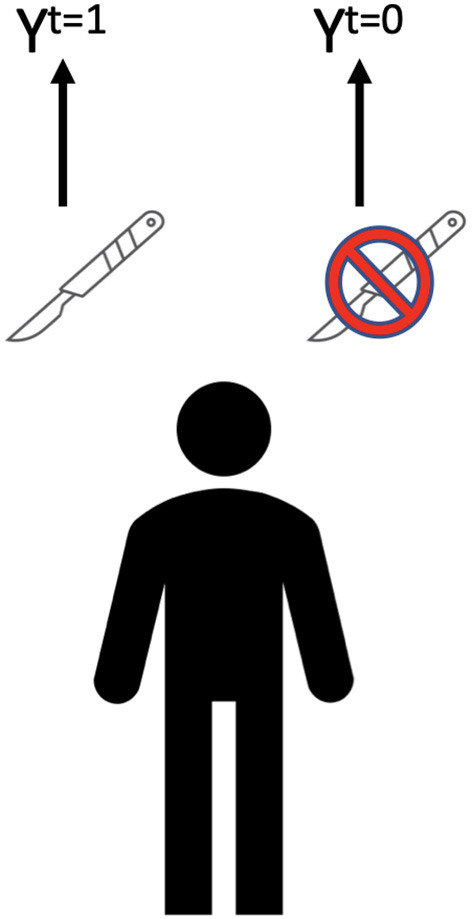
Outcomes following back surgery. Y^t = 1^ = outcome with, Y^t = 0^ = outcome without.

A key goal in assessing treatments for cLBP is to compare what outcome a patient would have if an intervention was delivered to what it would be if it was withheld. For example, cLBP is often treated with surgery (Figure 2). If the outcomes differ between surgery and no surgery than surgery has a causal effect on the outcome. This can be expressed as E (causal effect), of a given intervention T for a given outcome Y (E = Y^t = 1^ – Y^t = 0^). The fundamental problem of causal inference is that the individual causal effect can rarely be defined, as each individual only experiences one outcome (the exceptions are crossover experiments, such as n-of-1 trials) (Robins and Hernan, 2020). All of the counterfactual outcomes (i.e., the outcomes that would have occurred under the opposite treatment assignment, also known as potential outcomes) are missing. Therefore, causal effect is estimated at a population level.

To define the average causal effect in a population three pieces of information are needed for each individual—whether an intervention took place, what the outcome was if it did, and what the outcome would be if didn't (i.e., the counterfactual or potential outcome). Hypothetical data from ten individuals in a sample population is in Table 2. For each subject only one outcome can be observed (Ya = 1 is the outcome if the intervention took place, and Ya = 0 is the outcome if it didn't). However, the missing values in the sample population can be inferred from the known values, allowing an average causal effect to be estimated. In general terms there are three methods for filling in these missing values-randomization, an instrumental variable design, and a confounder control design (Matthay and Glymour, 2020).

**Table 2:** Hypothetical outcome data from a random sample.

**ID**	**A**	**Ya = 1**	**Ya = 0**
1	0	?	Known
2	0	?	Known
3	0	?	Known
4	0	?	Known
5	1	Known	?
6	1	Known	?
7	1	Known	?
8	0	?	Known
9	0	?	Known
10	1	Known	?

?, Unknown.

Mendelian randomization (MR) uses genetic variants as instrumental variables for estimating the causal effects of a risk factor on an outcome (Elgaeva et al., 2020). Using this method body mass index (BMI), fewer years of schooling, smoking, greater alcohol consumption, and depression have all been shown to be causes of low back pain (Elgaeva et al., 2020; Williams et al., 2022).

**Figure 3:**
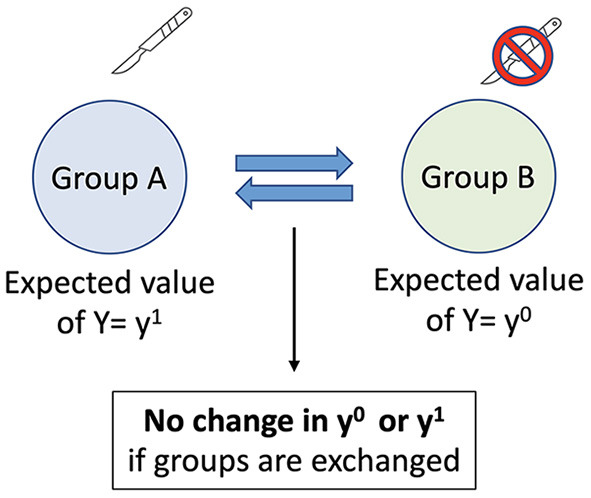
Example of Exchangeability. y^1^ = outcome, y^0^ = potential outcome.

**Figure 4:**
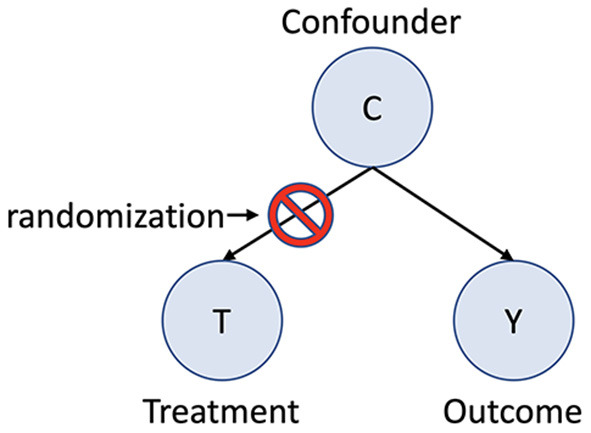
Example of Confounding Variable. C is a common cause of T and Y.

In a randomized experiment missing values—i.e., the potential or counterfactual outcomes- occur by chance, assuming a properly executed study (Robins and Hernan, 2020). This is because in a randomized trial the treatment assignment occurs by chance, and the outcome only varies because of the assigned treatment. Since the only difference between the two groups is the treatment assignment, which occurs by chance, the groups are exchangeable (Figure 3). The average causal effect can then be determined by using the mean values for the outcome with and without treatment: the average causal effect = (mean Y^t = 1^) – (mean Y^t = 0^). Exchangeability in randomized trials is assured because confounding bias has been eliminated due to random treatment assignment. Confounding bias occurs when a third factor is a common cause of both the treatment assignment and the outcome. For example, in the absence of randomization severe pain at baseline may be a common cause of both an assignment to an intervention as well as the outcome. Since one group now has more severe pain exchanging the treatment group with the control group will give different results. Randomization prevents confounding bias by blocking the effect of confounders on the treatment assignment (Figure 4), ensuring exchangability (Matthay and Glymour, 2020).

While randomization yields the most accurate estimations of causal effects, RCT's are frequently are not feasible for answering causal questions. An instrumental variable is an external factor that determines the chance of an exposure, but has no mechanism to influence the outcome except *via* the exposure. Instrumental variable study designs are conceptually like randomization, as individuals who are otherwise very similar receive different exposures (Matthay and Glymour, 2020).

As with randomization, a key assumption for instrumental variable designs is exchangeability—the assumption that an exposure is unrelated to the potential outcomes. If there is no instrumental variable that satisfies this assumption a confounder-control approach may be preferred (Matthay and Glymour, 2020). Confounder-control studies estimate the causal effect of interest by adjusting for a sufficient set of observed variables to control for confounding. The key assumption becomes conditional exchangeability; i.e., exchangeability is fulfilled after controlling for a set of measured covariates.

Confounder-control approaches rely on identifying, measuring, and correctly adjusting for a sufficient set of confounders. Multiple factors- such as patient and provider characteristics, socioeconomic parameters, environmental and geographical constraints- are potential sources of confounding in observational data (Velentgas et al., 2013). Identifying confounders is particularly challenging for complex conditions with many variables and uncertain (or unknown) causal relationships, such as cLBP In complex conditions many of the variables may be difficult to define, much less measure. A good example is pain, which is a multi-faceted perception that is unique to each individual. Whether the various instruments used to assess pain accurately enough capture the underlying construct is an open question. Furthermore, relationships between variables can vary according to context, and over time.

**Figure 5:**
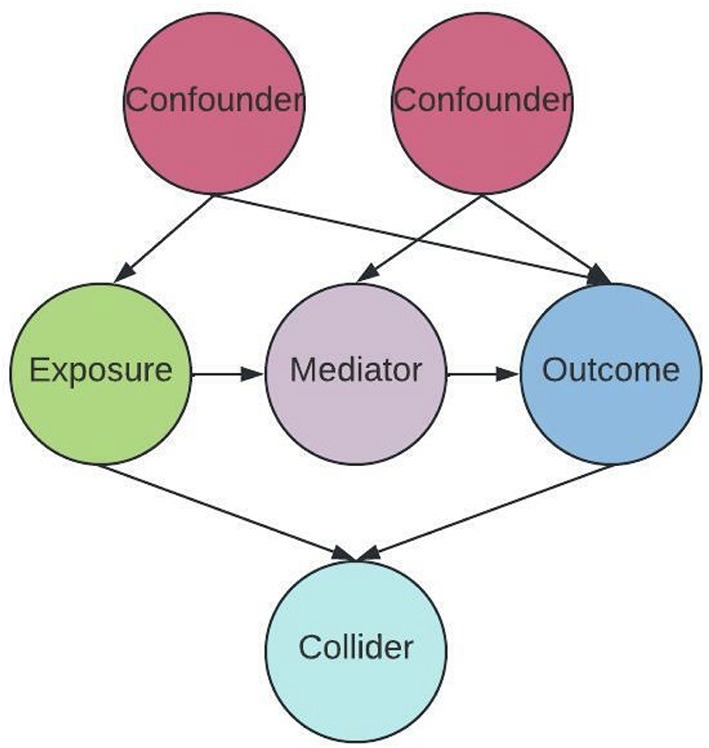
Paths in a Causal Directed Acyclic Graph (DAG). Circles are variable types, arrows indicate direction of effect.

The relationships between an exposure (such as a treatment), an outcome, and co-variates can be demonstrated in a directed acyclic graph (DAG) (Figure 5). Causal DAGs are models that depict assumptions about the causal structures linking variables. Differentiating confounders from other co-variates- notably mediators and colliders- is critical, as adjusting for other co-variates may increase bias, jeopardizing conditional exchangeability (Velentgas et al., 2013). Mediating variables explain the process that leads from an exposure to an outcome- they are part of the causal pathway. In Figure 4 the path from exposure to outcome is causal, *via* a mediator (Åkerblom et al., 2015). Confounding can affect both exposure-outcome relationships as well as mediator-outcome relationships. Colliding occurs when two independent causes have a common effect (Velentgas et al., 2013). For example, cLBP-related disability may result from physical impairment related to a peripheral biologic factor. However, it can also occur due to an independent cause, such as adverse effects of cLBP on affect and motivation.

Confounder control approaches in cLBP research have primarily been used to identify the mediators between exposures and outcomes, using a method known as mediation analysis. Most of this work has focused on the role of psychological factors as mediators (Lee et al., 2016; Sisco-Taylor et al., 2021; Joyce et al., 2022). The methodological quality of cLBP mediation analysis research is generally poor (Lee et al., 2016). The existing evidence indicates that psychological factors mediate the effects of psychological treatments, but not exercise-based treatments (Lee et al., 2016; Joyce et al., 2022). Mediation analysis has also found that psychological factors mediate the pain-disability relationship, but only explain 20–33% of the total effect. Identifying causal relationships between between exposures and cLBP outcomes, including the role of mediators, using more rigorous confounder-control methods is critical for optimizing diagnostic and therapeutic strategies.

In addition to conditional exchangeability two important assumptions for confounder-control designs are positivity and a stable unit treatment value (SUTV) (Matthay and Glymour, 2020). Positivity requires that for each subgroup of individuals defined by a covariate stratum (e.g., every combination of possible covariate values) exposure to the intervention is possible. A SUTV assumption means that all versions of the treatment have the same effect (i.e., any differences in the versions of the treatment an individual may have received are insufficient to alter the outcome), and that each individual's outcome is unaffected by the treatments that others may have received.

Once a sufficient set of confounders is identified the goal of the statistical analysis is to reduce confounding by breaking the association of the confounders with the outcome (e.g., regression adjustment); breaking the association of the confounders with the exposure (e.g., matching, adjustment, or weighting based on propensity scores); or breaking both the association with the exposure and the outcome (e.g., doubly robust methods) (Matthay and Glymour, 2020). These methods all allow comparisons within subgroups that have balanced covariates, such that the covariates cannot bias the treatment-outcome association. The validity of the causal estimates based on these methods relies on appropriate statistical inference, which includes considering random error, power, correct specification of the statistical model, accounting for multiple testing, and adjusting for differential loss to followup (Matthay and Glymour, 2020).

### Causal discovery

**Figure 6:**
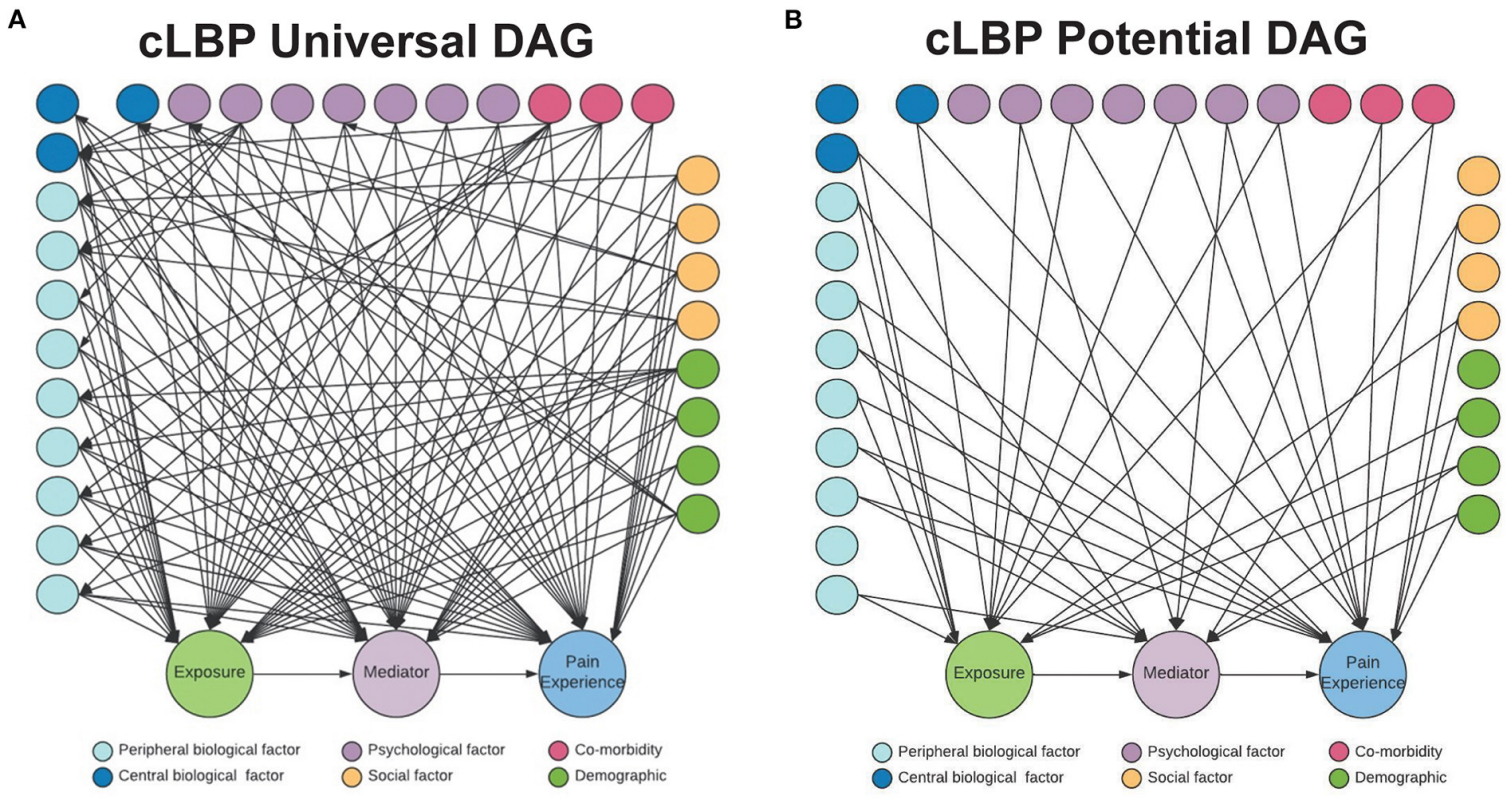
cLBP potential causal DAG. **(A)** Constraint-based causal algorithms begin with undirected graph, with all variables connected. **(B)** A potential causal DAG in which edges are pruned and a more parsimonious causal graph is created, that can then be furthered tested and refined.

Assumptions about causal relationships, as depicted in a causal DAG, are generally based on domain knowledge about the data generating process, which can incorporate prior research and/or or expert judgement. However, domain knowledge is rarely sufficient to completely characterize causal relationships (Velentgas et al., 2013). Given the large number of risk factors- from biological, psychological, and social domains- associated with cLBP, and the limited knowledge about causal relationships, a causal DAG representing potential relationships between risk factors and outcomes must necessarily be extremely complicated, and therefore of little practical value (Figure 6) (Elgaeva et al., 2020; Williams et al., 2022).

An alternative to using domain knowledge to identify causal relationships is causal discovery, a purely data-driven approach that uses artificial intelligence and high dimensional datasets to empirically select variables based on statistical associations (Velentgas et al., 2013). Confounders identified using causal discovery can then be used in statistical models that employ confounder-control approaches for identifying causal relationships.

Different causal machine learning algorithms approach the data with different initial assumptions, and some are better suited to different types of data (e.g., categorical, ordinal, vs. numeric; static vs. timeseries) than others. Causal machine learning algorithms fall broadly in to two categories: constraint-based, and score-based.

**Figure 7:**
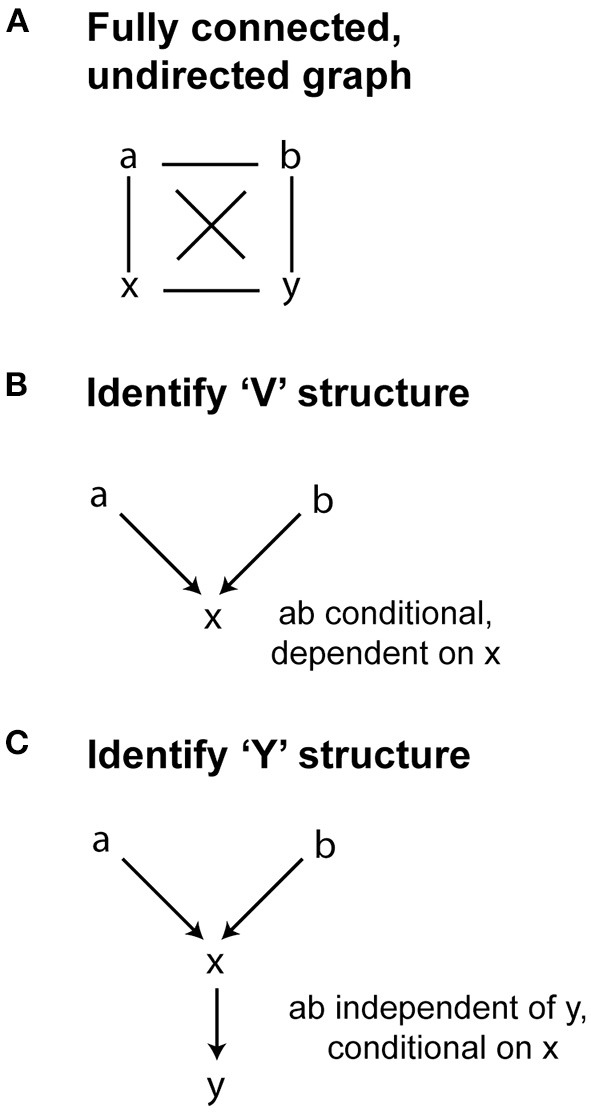
Fast Causal Inference Algorithm. **(A)** FCI begins with undirected graph. **(B)** FCI then removes edges and identifies possible “V” structures, where two variables are condtionally dependent on a third (colliders). **(C)** FCI also mitigates confounder bias by identifying “Y” structures, where two variables (a and b) are found to be independent of a third (y), conditional on a fourth variable x.

Constraint-based causal discovery methods test the conditional independence of links between data elements represented in a causal directed acyclic graph (DAG). Two common constraint-based methods are PC and Fast Causal Inference (FCI) (Glymour et al., 2019). PC assumes that there is no confounder, and its discovered causal information is asymptotically correct. FCI gives asymptotically correct results even in the presence of confounders (Glymour et al., 2019). FCI is initially defined by a causal graph in which all variables are connected, but with no directionality. The algorithm then systematically removes connections (or *edges*) that exist between variables that are conditionally independent. Using the remaining edges, FCI is designed to then identify two types of structures: colliders (or “V” structures) and “Y” structures. In “V” structures, two variables are unconditionally independent, but can become dependent conditional on a third variable (Shen et al., 2020). The “Y” structure is defined by 2 variables independent of a third, but conditional on a fourth variable (Figure 7). For instance, let's assume a and b cause x, and x in turn causes y. In a “Y” structure, a and b are independent of y, conditional on x. This identification of structures is a unique aspect of FCI, in that the conditional independence defined by this structure helps to rule out variables that induce dependence between otherwise independent pairs of variables (Shen et al., 2020).

There are three main assumptions underlying the constraint-based PC method (a) causal sufficiency assumption (b) causal Markov assumption and (c) faithful assumption. The causal sufficiency assumes the absence of a common un-observed cause of two or more nodes in a causal DAG. The Markov and faithful assumption relies on the notion that each variable in the causal DAG is independent with an independent probability distribution (Spirtes and Glymour, 1991; Zhang, 2008; Colombo et al., 2012). When taken together, these assumptions imply that the models induced by the data generating probability distribution and the causal DAG represent the same conditional independence statements.

The constraint-based algorithms such as basic PC algorithms for the observation data relies on the causal sufficiency, Markov, and faithful independent distributions to learn a causal DAG with conditionally independent nodes (Margaritis and Thrun, 1999). The FCI algorithms additionally can model the latent confounders, and finds the ancestral relationships between the measured variables (up to Markov equivalence). While informative, this approach is not able to infer the underlying causal DAG from the observational data (Spirtes et al., 2001). Despite recent successes in scaling to large numbers of variables, oth the PC and FCI algorithms, have historically been limited in their use on a large-scale observation data due to the requirement of search exponential space for all possible causal structures include those with the latent information by accounting for interactions and checking for conditional independence between the data elements (Silander and Myllymaki, 2012). Many improvements have been proposed to overcome the limitations of PC and FCI algorithms to enable causal discovery from the observational data. To this end, algorithms such as greedy fast causal inference methods have been proposed that combine the search criteria from greedy equivalence search with FCI algorithms (Spirtes et al., 2001).

In contrast with FCI, Fast Greedy Equivalence Search (FGES) is an optimized version of Greedy Equivalence Search that starts with a graph with no edges at all (Chickering, 2003). The algorithm then works iteratively to introduce dependencies in a way that is designed to optimize the model fit (without overfitting). This goodness-of-fit can be measured by a number of information criteria [e.g., Bayes Information Criteria (BIC), where a lower value indicates a better fit]. Given that the FGES model is specified using information criteria scores, this approach is referred to as a “score-based” algorithm.

Causal discovery methods are powerful tools, that overcome the limitations of empirically-driven methods (such random forest or LASSO) that are generally unable to distinguish between genuine confounders and other variable types, which may introduce bias (Velentgas et al., 2013). While causal discovery methods require making several assumptions about the structure of the data (which may affect the validity of the results), they can be applied to complex datasets to aid knowledge discovery (Spirtes et al., 2001; Glymour et al., 2019). In the next section we will explore how these methods may be applied to the complex problem of cLBP.

### Applying causal discovery models to cLBP research

Application of causal modeling to cLBP is in its infancy, with most prior work focusing on RCTs and traditional statistical inference. However, the complexity and multifaceted nature of cLBP makes it a strong candidate for the application of more advanced causal models. Identification of causal relationships between variables in the BACPAC multi-modal dataset using causal discovery algorithms can help to solve important applied clinical problems.

Applied problem #1: What mediates the effects of treatments for cLBP?

Mediation analysis decomposes the total exposure-outcome effect into a direct effect and an indirect effect through a mediator variable (Rijnhart et al., 2021). Traditional approaches to meditation analysis, such as the familiar Barron and Kenney method (Baron and Kenny, 1986), have significant limitations, as they are prone to biased estimates if there is mediator-outcome confounding, exposure-mediator interactions, non-continuous mediator and outcome variables, or multiple mediators (Rijnhart et al., 2021). Causal mediation analysis, which is based on a counterfactual framework that requires understanding causal relationships, overcomes these limitations. Applying causal mediation analysis to the BAPCPAC dataset would improve understanding of how cLBP treatments work and why they fail, leading to improved treatment strategies.

Applied problem #2: Can subgroups of cLBP patients with improved treatment effects be identified?

Identifying subgroups of patients with enhanced effects to treatments from observational data is challenging (Kent et al., 2019). One method for doing so is to define a conditional average treatment effect (CATE), using a potential outcomes framework that contrasts outcomes conditioned on covariates (Yadlowsky et al., 2020). As with other causal inference methods an important assumption in defining CATE is conditional exchangeability (Robertson et al., 2020). While unmeasured confounding is always a threat to exchangeability, the more complete the knowledge about the causal structure of dataset, the less this threat will be.

## Discussion

Limited understanding of the causal relationships underlying the associations between risk factors and cLBP is a major reason for failed treatments. Failed treatments for cLBP are associated with a variety of neuropsychiatric co-morbidities including anxiety, depression, substance abuse, and opiod addiction. Applying causal discovery methods to the rich, multidimensional BACPAC dataset, harmonized across multiple centers employing a variety of study designs, will clarify causal relationships, enabling innovative approaches to improving therapeutic strategies. Future work to apply the advanced causal models discussed above to this dataset will be important to drive knowledge discovery. Upon successful causal model development, a number of challenges will still need to be met. Validation of the clinical utility of the model will be key, wherein clinical trials will be designed to test hypotheses arising from causal mediation analyses, and to further test clinical utility hypotheses that are generated by causal machine learning results. Similarly, a model that fits your own data may be useful, but transportability of the model to other datasets is necessary for a truly useful model. This can be a difficult prospect if there are relatively few external sources of data with a similar breadth and/or depth as the one from which the model was built. In the case of BACPAC and similar multisite endeavors, one may consider keeping one site as a holdout on which to validate the model. Despite these challenges, there remains an opportunity to develop a model that can help researchers better understanding causal factors for cLBP, and guide clinical practice in the future.

## Author contributions

CO'N, JH, and AF conceived of review. CO'N, JH, and RV wrote initial manuscript. JH, RV, AF, SM, AB, AG, CH, and CO'N edited and revised final manuscript. All authors contributed to the article and approved the submitted version.
